# Structural connectivity at term equivalent age and language in preterm children at 2 years corrected

**DOI:** 10.1093/braincomms/fcae126

**Published:** 2024-04-10

**Authors:** Maria E Barnes-Davis, Brady J Williamson, Julia E Kline, Beth M Kline-Fath, Jean Tkach, Lili He, Weihong Yuan, Nehal A Parikh

**Affiliations:** Perinatal Institute, Cincinnati Children’s Hospital Medical Center, Cincinnati, OH, USA; Department of Pediatrics, University of Cincinnati College of Medicine, Cincinnati, OH, USA; Department of Radiology, University of Cincinnati College of Medicine, Cincinnati, OH, USA; Perinatal Institute, Cincinnati Children’s Hospital Medical Center, Cincinnati, OH, USA; Department of Radiology, University of Cincinnati College of Medicine, Cincinnati, OH, USA; Department of Radiology, Imaging Research Center, Cincinnati Children’s Hospital Medical Center, Cincinnati, OH, USA; Department of Radiology, University of Cincinnati College of Medicine, Cincinnati, OH, USA; Department of Radiology, Imaging Research Center, Cincinnati Children’s Hospital Medical Center, Cincinnati, OH, USA; Department of Radiology, University of Cincinnati College of Medicine, Cincinnati, OH, USA; Department of Radiology, Imaging Research Center, Cincinnati Children’s Hospital Medical Center, Cincinnati, OH, USA; Department of Radiology, University of Cincinnati College of Medicine, Cincinnati, OH, USA; Cincinnati Children’s Hospital Medical Center, Pediatric Neuroimaging Research Consortium, Cincinnati, OH, USA; Perinatal Institute, Cincinnati Children’s Hospital Medical Center, Cincinnati, OH, USA; Department of Pediatrics, University of Cincinnati College of Medicine, Cincinnati, OH, USA

**Keywords:** prematurity, connectivity, language, diffusion MRI, development

## Abstract

We previously reported interhemispheric structural hyperconnectivity bypassing the corpus callosum in children born extremely preterm (<28 weeks) versus term children. This increased connectivity was positively associated with language performance at 4–6 years of age in our prior work. In the present study, we aim to investigate whether this extracallosal connectivity develops in extremely preterm infants at term equivalent age by leveraging a prospective cohort study of 350 very and extremely preterm infants followed longitudinally in the Cincinnati Infant Neurodevelopment Early Prediction Study. For this secondary analysis, we included only children born extremely preterm and without significant brain injury (*n* = 95). We use higher-order diffusion modelling to assess the degree to which extracallosal pathways are present in extremely preterm infants and predictive of later language scores at 22–26 months corrected age. We compare results obtained from two higher-order diffusion models: generalized q-sampling imaging and constrained spherical deconvolution. Advanced MRI was obtained at term equivalent age (39–44 weeks post-menstrual age). For structural connectometry analysis, we assessed the level of correlation between white matter connectivity at the whole-brain level at term equivalent age and language scores at 2 years corrected age, controlling for post-menstrual age, sex, brain abnormality score and social risk. For our constrained spherical deconvolution analyses, we performed connectivity-based fixel enhancement, using probabilistic tractography to inform statistical testing of the hypothesis that fibre metrics at term equivalent age relate to language scores at 2 years corrected age after adjusting for covariates. Ninety-five infants were extremely preterm with no significant brain injury. Of these, 53 had complete neurodevelopmental and imaging data sets that passed quality control. In the connectometry analyses adjusted for covariates and multiple comparisons (*P* < 0.05), the following tracks were inversely correlated with language: bilateral cerebellar white matter and middle cerebellar peduncles, bilateral corticospinal tracks, posterior commissure and the posterior inferior fronto-occipital fasciculus. No tracks from the constrained spherical deconvolution/connectivity-based fixel enhancement analyses remained significant after correction for multiple comparisons. Our findings provide critical information about the ontogeny of structural brain networks supporting language in extremely preterm children. Greater connectivity in more posterior tracks that include the cerebellum and connections to the regions of the temporal lobes at term equivalent age appears to be disadvantageous for language development.

## Teaser

Barnes-Davis *et al*. investigate ontogeny of brain networks supporting language in extreme prematurity using two higher-order analysis pipelines for diffusion MRI. They report connectivity between temporal lobes in infancy (bypassing the body of the corpus callosum and involving the cerebellum) negatively relates to language performance at 2 years.

## Graphical Abstract

**Graphical Abstract:**
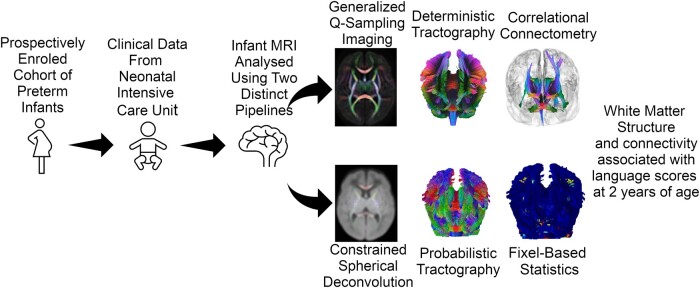

## Introduction

Approximately 10% of children born in the USA and worldwide are preterm at birth.^1,2^ However, the impact of this public health crisis is felt beyond the immediate families of preterm children, not only in terms of mortality and long-term morbidity but also in terms of reduced societal attainments and increased health care expense.^3-6^ While survival at the youngest gestational ages (GAs) is improving, rates of neurodevelopmental impairment are not showing similar gains.^7-9^ Strong associations between preterm birth and poor neurodevelopmental outcomes remain, with infants born extremely preterm (EPT, born at <28 weeks completed gestation) at greatest risk.^7,8,10,11^

Adverse neurodevelopmental outcomes in EPT have been linked to brain injury and dysmaturation.^12-16^ Initial reports on aberrant brain development in prematurity focused on discrete injury, such as parenchymal lesions, periventricular leukomalacia (PVL) and intraventricular haemorrhage (IVH).^17,18^ Investigation into the neural dysmaturation of EPT, so-called ‘encephalopathy of prematurity’ involving destruction and aberrant development of neuronal bodies and axons following discrete injury, initially relied on animal models and post-mortem examination of the brain.^18^ Over the past 40 years, advances in non-invasive neuroimaging have propelled the field of neonatal neurology using non-invasive approaches studying brain function [such as magnetoencephalography (MEG) and functional MRI] and brain structure (structural and diffusion MRI), and this approach is now preferred.^12,18-20^

Brain injury on cranial ultrasound has been related to motor and cognitive outcomes at 2 years corrected age and beyond.^21-23^ However, it is increasingly recognized that dysmaturation can, and does, occur in the preterm brain, even in the absence of a clinically recognized overt injury such as PVL or IVH.^12,18,24-26^ The third trimester of gestation is an active period of *in utero* development for both the cerebrum and the cerebellum.^27^ Developing neuronal progenitor cells can be adversely impacted by prematurity, with the timing of insult resulting in decreased surface area of the cortical grey matter (if insult occurs before 25 weeks) and/or decreased cortical thickness (if insult occurs after 25 weeks).^28,29^ If the last trimester of gestation is spent *ex utero*, aberrant development can include factors such as inflammation and hypoxic–ischaemic injury, adversely impacting preoligodendrocytes and microglia and leading to increased programmed cell death, aberrant myelination and gliosis.^18,30,31^ This altered development of cerebral white matter can be appreciated on diffusion-weighted MRI (dMRI).^22,32,33^ Sophisticated analytical techniques can be used to interrogate dMRI data, and such data are predictive of cognitive outcomes and motor disorder and disability.^13-16,33-38^

However, language outcomes are harder to predict. Studies have shown that structural MRI obtained at term equivalent age (TEA) and 2-year language composite scores from the widely used Bayley Scales of Infant and Toddler Development (BSID) account for a low proportion of the variance for language functioning later on in childhood.^39-41^ Children born EPT are at higher risk for language difficulties than term children.^41-46^ Results regarding the developmental trajectory of language delays or deficits have been conflicting.^42,47-49^ Language is important not only for scholastic and professional attainment but also for development of relationships with peers and caregivers and resulting quality of life.^50-52^ Language difficulties might contribute to other known comorbidities of prematurity, such as increased risk for abuse and non-accidental trauma, decreased likelihood of having significant relationships as adults and decreased quality of life.^53-57^

Previously, we demonstrated that functional hyperconnectivity, as indexed by functional MRI-constrained MEG, in EPT children aged 4–6 years versus their term counterparts (TC) was positively associated with language scores for the EPT group exclusively.^24,58,59^ In the same cohort of children, we interrogated the structural connectivity underlying this functional hyperconnectivity using higher-order tensor-free analysis of dMRI data, a specialized approach developed to better enable the evaluation of connectivity in voxels containing complex fibre orientations, such as crossing fibres (now known to impact over 90% of the human brain).^60^ Using this state-of-the-art structural connectometry pipeline, we found that the functional hyperconnectivity between bilateral temporal regions in the EPT group was supported by increased structural connectivity in extracallosal pathways (bypassing the body of the corpus callosum), including the cerebellar white matter, middle cerebellar peduncles, corticothalamic white matter, corticopontine white matter, corticospinal white matter and the external capsules.^25^ Connectivity in this extracallosal pathway was positively associated with language scores for the EPT group but not for the TC group.^25^

The aim of this current study is to investigate when this extracallosal hyperconnectivity becomes apparent in EPT children without significant brain injury. To do this, we leverage a well-characterized, regional, prospective cohort study of 350 very preterm (VPT, <32 weeks completed gestation) infants born in the greater Cincinnati area and followed with multimodal neuroimaging at TEA and longitudinal neurobehavioral assessments as part of the Cincinnati Infant Neurodevelopment Early Prediction Study (CINEPS). The overarching goal of the CINEPS study is to prospectively obtain neonatal anatomical, functional and diffusion MRI to detect subtle brain abnormalities and predict, soon after birth, long-term neurodevelopmental outcomes. Published works from the CINEPS study have demonstrated the predictive potential of neuroimaging markers including diffuse white matter abnormality and structural connectivity.^14,36-38,61^ As a part of the longitudinal study, participants undergo T_1_-weighted, T_2_-weighted and multishell high-angular-resolution diffusion imaging (HARDI) that is amenable to higher-order tensor-free dMRI models. In this report, we will use two such models: (i) q-space diffeomorphic reconstruction (QSDR) based on generalized q-sampling imaging (GQI)^62,63^ followed by deterministic tractography and correlational connectometry^64^ and (ii) constrained spherical deconvolution (CSD)^65^ followed by probabilistic tractography and connectivity-based fixel enhancement (CBFE).^66^

This study is a secondary analysis of the established CINEPS cohort, using the subgroup that was born EPT without significant brain abnormality. We tested the following specific hypotheses:

1. Structural connectometry analysis of dMRI obtained at TEA will demonstrate an extracallosal pathway (bypassing the body of the corpus callosum) connecting the right and left temporal regions that will positively correlate with language scores on the BSID performed at 2 years corrected age in infants born EPT with no known brain injury.
2. CSD analysis of dMRI with CBFE will relate to BSID language composite scores and provide complementary results to structural connectometry findings.

## Materials and methods

### Study design and participants

This is a secondary analysis of a prospective cohort study conducted at Cincinnati Children’s Hospital Medical Center (CCHMC) and four additional affiliated neonatal intensive care units (NICUs), including the University of Cincinnati Medical Center, Good Samaritan Hospital, Kettering Medical Center and St. Elizabeth’s Healthcare. From 2017 through 2019, 350 children born VPT were recruited from these five Level 3 or 4 NICUs in the Greater Cincinnati area. For this secondary analysis, we included only children born EPT (<28 weeks completed gestation) and without significant brain injury on their MRI as defined below. Inclusion criteria for the original cohort included GA ≤32 weeks and written informed consent of the parents. Exclusion criteria included known cyanotic congenital heart disease and genetic syndromes or congenital anomalies known to impact the CNS. Children were followed through 3 years corrected age to comprehensively assess neurodevelopmental outcomes. Full study procedures have been published elsewhere.^15,61^ The study was approved by the CCHMC Institutional Review Board that had a reciprocity agreement with the four affiliated NICUs and conforms to the US Federal Policy for the Protection of Human Subjects and the Declaration of Helsinki.

### Demographic, postnatal and neuropsychological assessments

Our primary neurobehavioral outcome of interest was the language composite score from the BSID.^40^ The BSID was administered by trained assessors. Children in the cohort were scheduled to participate in this assessment at 22–26 months corrected age. Relevant demographic and postnatal variables were collected prospectively using standard definitions,^15^ including GA, post-menstrual age (PMA) at time of imaging, corrected age at time of BSID assessments, sex, race, ethnicity, parental education level, parental income and global brain abnormality (GBA) score.^67,68^ A social risk score was calculated using family structure, parental education, occupation, family income and maternal age.^69^ Significant brain injury was defined as any PVL or severe IVH (greater than Papile Grade 2) reported in the clinical radiology reads of cranial ultrasounds performed serially in the NICU as part of routine clinical care.^70^

### MRI acquisition

MRI of the brain was obtained for all participants at TEA (39–44 weeks PMA) using a 3T Philips Ingenia scanner with a 32-channel adult head coil. Infants were scanned without sedation during natural sleep (using the feed and swaddle method)^71^ and were fitted with ear plugs and muffs to protect hearing. All scans were attended by a clinical research coordinator and MRI technician. For infants who were still admitted to the NICU and on positive pressure ventilation, an attending neonatologist (N.A.P., M.E.B.-D.), NICU nurse and respiratory therapist accompanied them to the imaging suite as well. 3D T_1_-weighted MRI was obtained using a magnetization-prepared rapid acquisition gradient echo (MP-RAGE) sequence [TR (repetition time)/TE (echo time)/TI (inversion time) = 8.5/3/1610 ms and resolution 1.0 × 1.0 × 1.0 mm]. High-resolution T_2_-weighted MRI was obtained with TR/TE = 19 377/166 ms and resolution 1.0 × 1.0 × 1.0 mm. HARDI was obtained using *b*-values of 800 and 2000 s/mm^2^. Only the b2000 data are analysed here. Parameters for the multiband acquisition were TR/TE = 5073/88 ms, a resolution of 2.0 × 2.0 × 2.0 mm, 68 directions and an acceleration factor of 4. The entire structural acquisition took <15 min. All scans were read by a board-certified paediatric neuroradiologist. This same radiologist, blinded to clinical data, determined GBA scores using a standard system developed by Kidokoro *et al.*,^67^ which assessed the cortical and deep grey matter, cerebral white matter, ventricles and cerebellum. Higher scores indicate more abnormality (<4, no abnormality; 4–8, mild abnormality; 8–11, moderate abnormality; >11, severe abnormality).^67^

### Statistical analyses

#### QSDR and connectometry

##### Justification

QSDR is a model-free, quantitative, higher-order technique of analysing dMRI data that is better able to resolve incoherent fibre orientations (crossing fibres) than traditional tensor-based techniques (i.e. diffusion tensor imaging or DTI).^62^ This is important, as over 90% of the voxels in the brain contain crossing fibres.^60,72^ Following QSDR, we use deterministic tractography to perform correlational connectometry analysis, a method less sensitive to false positives than other analytical techniques employed in the field.^73^ Additionally, we conducted these analyses on the whole-brain level, not constrained to specific regions of interest or white matter tracts based on adult models of language networks. Our data-driven preprocessing pipeline and application of these analytical techniques to children born EPT have been published in full previously.^25,74^

##### Preprocessing

In brief, diffusion data for QSDR and connectometry analyses were preprocessed using TORTOISE (Tolerably Obsessive Registration and Tensor Optimization Indolent Software Ensemble).^75^ This included denoising,^76^ removing Gibbs-ringing artefact^77^ and correcting motion, eddy and geometric distortions.^75,78^ Original diffusion gradient vectors were rotated at each relevant step in the preprocessing pipeline. The T_2_-weighted scan was skull stripped in AFNI.^79^ The skull-stripped image was then used for rigidly registering the dMRI data and the B-spline geometric distortion correction. Preprocessed diffusion MRI data were then imported into DSI Studio (http://dsi-studio.labsolver.org). During quality control, diffusion volumes with a neighbouring correlation coefficient of <0.9 were removed.^80^ Participants were excluded if >10% of volumes were removed.

##### Diffusion reconstruction and connectometry database creation

GQI, which estimates the spin distribution function (SDF) from the underlying diffusion signal using a Funk–Radon transform,^63^ was used to reconstruct the signal for each participant and generate quantitative anisotropy (QA) maps. Importantly, each data set was checked for accurate diffusion gradient orientation using an automated method (--check_btable in DSI Studio) to correct any discrepancies between TORTOISE and DSI Studio. QA maps from a previously published study-specific template^81^ were averaged to create a study-specific template using ANTs multivariate template reconstruction.^80^ We then used QSDR^62,63^ to obtain each participant’s SDFs in template space. A more detailed description of this methodology is provided in our previous work.^25^ The connectometry database was created by aligning all SDFs on the participant level. The spatial correlation coefficient assessed the quality of the registration. If any participant had a normalized average SDF value > 2 SDs above the rest of the participants, that child’s data set was excluded. However, no participants required exclusion at this step.

##### Connectometry analysis and relation to developmental outcome

Structural connectometry was performed at the whole-brain level to investigate the degree to which white matter connectivity at TEA related to BSID scores at 2 years corrected age. In this analysis, we used a *t*-threshold of 2.0, so that only SDF values with a moderate to high correlation were retained. We performed two iterations of topology-informed pruning of tracts, used a length threshold of 20 voxels (40 mm) and set a false discovery rate (FDR) of 0.05 using 4000 permutations. QA was normalized for the SDFs to account for any changes due to PMA at time of MRI scan. Structural connectivity (indexed by QA) at TEA was related to standardized BSID scores for the language composite (primary outcome) and for the cognitive and motor composites (secondary outcomes, see Supplementary material). In the structural connectometry analysis, PMA at time of scan, sex as a biological variable, social risk score and GBA score were included as nuisance regressors in the model. To assess the nature of the relationship between significant tracts from connectometry and language performance, mean normalized QA, adjusted for the other covariates, in significant tracks was correlated (Spearman’s rho) with language performance to assess the proportion of variance accounted for by the model.

#### CSD and CBFE

##### Justification

Spherical deconvolution is multicompartmental, model-based method used to address the problem of incoherent fibre orientations in voxels of dMRI, thereby improving upon older, tensor-based metrics. It is not reliant on the assumption that there is one direction (or tensor) of maximal diffusion. In this method, the fibre orientation distribution (FOD) is calculated for each voxel, assuming the diffusion signal from fibre bundle orientations in multiple directions can be modelled as a single function.^65^ CSD is a constrained form of spherical deconvolution in which artefactual negative orientations are limited, thereby reducing noise and improving the ability of tractography to resolve voxels with crossing fibres.^82^ Resultant diffusion metrics are fibre density (FD), fibre cross-section (FC) and the fibre density and cross-section product (FDC). Importantly, unlike QSDR, this model is not based in q-space (which provides an estimate of the spin propagation and distribution function) but instead focuses specifically on fibre orientations.^82^ Therefore, while related, CSD and QSDR methods might provide unique, complementary information.

##### Preprocessing

Our CSD preprocessing and analysis approach has been explained in full previously.^81,83^ In brief, diffusion MRI data were preprocessed in MRtrix3 (https://www.mrtrix.org/), an open-source software package that includes standard FSL functions (http://fsl.fmrib.ox.ac.uk/fsl/fslwiki/) to prepare data for CSD analysis.^84^ This includes principal component analysis, denoising, correction for Gibbs-ringing artefacts, motion correction, eddy current reductions, bias field correction, global intensity normalization and upsampling to an isotropic resolution of 1.3 mm^3^.^81^ This preprocessing pipeline was chosen for this approach to abide by guidelines of the MRtrix documentation. However, we also compared the results of our analyses using each preprocessing workflow for each method to investigate if the choice of preprocessing options changed the results.

##### CSD

In MRtrix3, we used the CSD pipeline to model the white matter FOD on a voxel-by-voxel basis for each participant using the Tournier algorithm.^83^ We again used a previously published study-specific template.^81^ We then segmented the resultant FOD template to produce a group-level template.^66^ Participant-level FODs were warped, registered to the group template and segmented to generate ‘fixels’ (a fibre bundle element per voxel). This enables the Jacobean of the warp to be analysed to give an estimate of how much a given participant deviates from the group-level fixel template.

##### CBFE and relation to developmental outcomes

In the CBFE analysis, the fixels are ‘smoothed’ *post hoc* using information from the whole-brain tractography performed based on the CSD outputs (FD, FC and FDC) to identify connectivity between fixels that are likely to be structurally (anatomically) connected.^66^ After smoothing, we fit a covariate-corrected general linear model in each fixel of the white matter to test the hypotheses that there is a relationship between CSD outputs (FD, FC and FDC) and language outcome, allowing fixels to survive only if they pass family-wise error (FWE) correction. FWE rate correction was set at 0.05. The output of this model is not a connectivity metric, but rather the fixels that are correlated with language outcome after covariate correction and FWE.

## Results

### Demographics and neuropsychological assessments

Of the 350 children enroled in the CINEPS cohort, 120 were born EPT. Of these, 95 had no history of significant IVH or PVL. Of those 95, 4 were missing b2000 data and BSID scores and 4 were missing BSID scores only. Thirty-three had no neuroimaging data that passed quality control but had BSID scores. Fifty-four had complete neuroimaging and neuropsychological data sets, and 53 passed quality control. Thus, 53 children are included in the final analysis of this study. Demographic information and mean neuropsychological and GBA scores for these 53 children are shown in Table 1. Of note, 59% of the sample were female. The mean GA was 26.4 weeks with a SD of 1.4 weeks. The mean PMA at time of MRI scan was 43.1 weeks with a SD of 1.2 weeks. Regarding the social risk score, 11% of the sample had a score that was considered high risk. Additionally, we did assess clinical and demographic differences between EPT children who had complete neuroimaging (*n* = 54) and whose who did not have complete neuroimaging (*n* = 41). Infants who completed neuroimaging suitable for analyses were more likely to be female, of White/Caucasian race and had slightly lower GBA scores. These data are presented in Supplementary Table 1.

**Table 1:** Demographic, clinical and neurodevelopmental data (*n* = 53) for all participants

Gestational age (weeks)	26.4 (±1.4)
Birth weight (g)	872.0 (±237.3)
BSID language composite	91.3 (±15.0)
BSID cognitive composite	90.8 (±11.2)
BSID motor composite	91.5 (±10.6)
Global Brain Abnormality Score	6.2 (±4.4)
Post-menstrual age at MRI (weeks)	43.1 (±1.2)
Sex	
Female	31 (59%)
Male	22 (42%)
Race	
American Indian/Alaskan Native	1 (2%)
Black/African American	4 (8%)
White/Caucasian	38 (72%)
Multiple	5 (9%)
Decline to respond	5 (9%)
Mode of delivery	
Breech	1 (2%)
C-section	32 (60%)
Vertex	20 (38%)
SGA	4 (8%)
ANCS	50 (94%)
Magnesium	47 (89%)
High risk social	6 (11%)
Mild IVH	9 (17%)
HDP	19 (36%)
NEC	3 (6%)
Sepsis	12 (23%)
PDA	27 (51%)
Caffeine	53 (100%)
Severe BPD	18 (34%)
Severe ROP	9 (17%)

Continuous data presented as mean (SD). Categorical data presented as count (percentage). ANCS, antenatal corticosteroids; BPD, bronchopulmonary dysplasia; BSID, Bayley Scales of Infant and Toddler Development; C-section, Caesarean section; HDP, hypertensive disorders of pregnancy; IVH, intraventricular haemorrhage; PDA, patent ductus arteriosus; ROP, retinopathy of prematurity; SGA, small for gestational age.

### Structural connectometry

**Figure 1:**
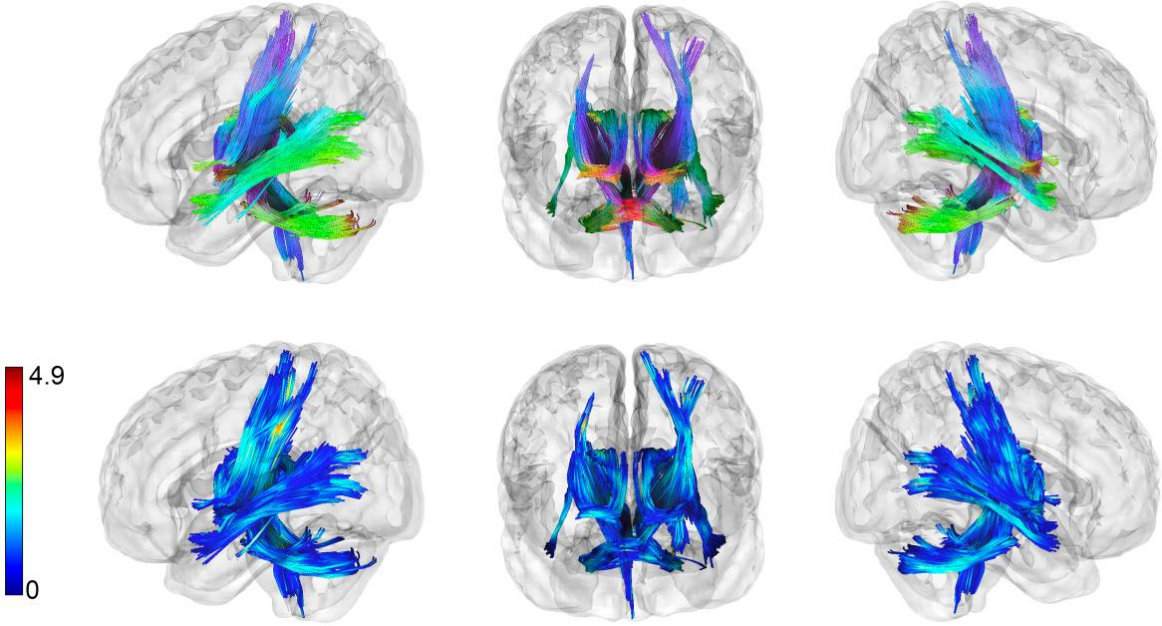
**Structural connectometry in EPT infants at TEA (preprocessed using TORTOISE).** Results of connectometry analysis relating white matter connectivity at TEA to language composite scores from the BSID. 3rd Edition (BSID) assessed at 2 years corrected age. Analyses preprocessed using the TORTOISE pipeline revealed tracks negatively (FDR *P* = 0.001) associated with BSID language scores while controlling for multiple comparisons and for the following confounding variables: PMA (PMA at time of MRI); sex; social risk score; and GBA score (*t*-threshold = 2.5, length threshold = 20 voxels, 2 pruning iterations, 4000 permutations). The *top* row is a figure colour coded by fibre orientation/direction, and the *bottom* row is colour coded by local *t*-statistic value of the underlying SDFs. Tracks negatively associated with language performance include bilateral CSTs, middle cerebellar peduncle, bilateral cerebellar tracts and posterior IFOF. Notably, all tracks shown must have first passed the *t* = 2.5 threshold for the connectometry analysis. *t*-score here is for the subsequent analysis after these tracks were selected. Strongest effects for tracks negatively associated with language are in the middle cerebellar peduncle and left CST and corticostriatal tracts.

**Figure 2:**
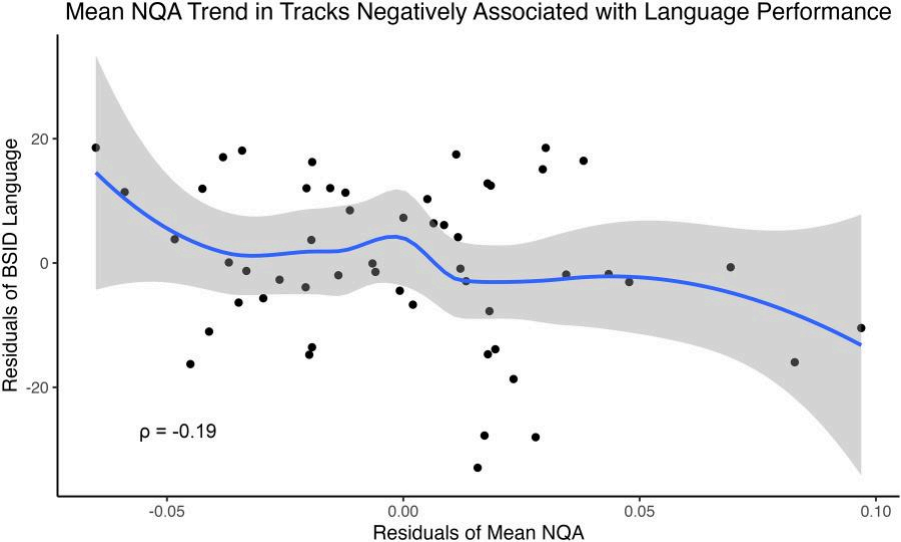
**Language scores plotted by mean normalized QA (NQA) residuals (preprocessed using TORTOISE).** Plots of BSID language scores by mean NQA values, adjusted for the partial effects of sex, GBA score, social risk score and PMA, for tracks negatively associated with BSID language in the connectometry analysis with preprocessing using TORTOISE. Correlation coefficient (Spearman’s rho) between adjusted mean NQA and BSID language is also shown. Tracks negatively associated with language performance show the most robust effect in the *bottom* half of NQA values.

Whole-brain connectometry analysis of dMRI at TEA showed widespread significant negative associations between structural connectivity (indexed by normalized QA) and language scores at 2 years corrected age for the EPT children (Fig. 1). Tracts showing a significant negative correlation with language include bilateral cerebellar white matter, middle cerebellar peduncles, bilateral tracks appearing to traverse the corticospinal and corticostriatal tracts, some tracks in the region of the posterior inferior fronto-occipital fasciculus (IFOF) and the posterior commissure (Fig. 1, top row). Visualization of the *t*-statistics for the underlying SDFs shows that the largest effect sizes for tracks negatively associated with later language outcome are the middle cerebellar peduncles and tracks in the region of the left corticospinal and corticostriatal tracts (Fig. 1, bottom row). Plots of the residuals from the linear model regressing normalized QA from significantly associated tracks in Fig. 1 against BSID language scores are shown in Fig. 2 with the positive and negative Spearman’s rho.

There were no significant results that survived correction for multiple comparisons for cognitive or motor outcomes. All results replicated when QSDR and connectometry were performed on data preprocessed using the MRtrix-specific pipeline (Supplementary Figs 1 and 2). The only differences were greater distribution of tracks that passed the statistical threshold and a slightly higher FDR-corrected *P*-value (0.008 versus 0.001 for TORTOISE preprocessed data).

### CBFE

CSD followed by CBFE of diffusion MRI data at TEA did not have any positive or negative associations that survived adjustment for covariates and correction for multiple corrections for FC, FD or FDC. These results also replicated when CSD and CBFE were performed on TORTOISE preprocessed data. This was true not only for association with BSID language scores but also for BSID motor and cognitive composite scores.

## Discussion

In this secondary analysis of a prospective, longitudinal multimodal neuroimaging study of outcomes of prematurity, we used two higher-order, tensor-free models of dMRI to investigate whether the extracallosal structural connectivity we have reported in well-performing EPT children^25,74^ is present at TEA and if these bilateral pathways that avoid the body of the corpus callosum are positively correlated with language as early as TEA. Our first hypothesis was that structural connectometry analysis of dMRI obtained at TEA in EPT children without significant brain injury would demonstrate an extracallosal pathway bypassing the body of the corpus callosum and supporting bilateral language areas and that structural connectivity at TEA would positively relate to language outcome at 2 years corrected age. This hypothesis was not confirmed, as we found that connectivity in extracallosal pathways was negatively associated with language scores at 2 years. Our second hypothesis was that simultaneous analysis of dMRI data using CSD and CBFE would provide metrics of white matter structural properties that are complementary to the QSDR and structural connectometry analyses. This was not confirmed, as our CSD and CBFE analyses yielded no significant results. This may be due to insufficient power due to our small sample size (but additional considerations are described below).

Our whole-brain structural connectometry analysis showed negative associations between structural connectivity at TEA and language scores at 2 years corrected age for the EPT children. This is not consistent with our studies relating white matter connectivity to language outcome in 4- to 6-year-old EPT children using similar methods.^25,74^ In our prior studies of preterm children at early school age, EPT children had decreased structural connectivity overall versus their term developmental comparisons; however, they did have increased structural connectivity in posterior cerebral pathways such as the IFOF, bilateral corticospinal tracts (CSTs), the splenium and genu of the corpus callosum, the bilateral posterior arcuate fasciculi, cerebellar peduncles and the cerebellum. This increased structural connectivity appeared to support increased functional connectivity during language tasks in fMRI.^24,59^ Additionally, when we limited our analyses to areas of the brain that showed significant activation during language tasks (including bilateral temporal lobes), our well-performing EPT group had positive correlations between language scores at 4–6 years of age and structural connectivity in bilateral corticospinal pathways, bilateral external capsules, bilateral cerebellar peduncles, posterior IFOF, splenium, cerebellar white matter and left corticothalamic and corticopontine pathways.^25,74^ Our EPT cohort with a history of language difficulties had positive correlations with language scores and more ‘typical’ areas. We concluded there were differential relationships between white matter connectivity and language performance between for EPT children with and without a history of language difficulty or disorder, wherein connectivity in superior, anterior tracts is associated with language difficulty or disorder in EPT children while connectivity in inferior, posterior tracts leads to better performance without the need for speech and language services in the context of prematurity.

In the current analysis, it is interesting to note that structural connectivity at TEA in some tracks showed a significant negative correlation with language scores at 2 years, including bilateral cerebellar white matter and middle cerebellar peduncles, some shorter tracks in the region of the posterior IFOF and some tracks appearing to traverse the corticospinal and corticostriatal tracts. These negative correlations between structural connectivity of the cerebellum at TEA and language scores at 2 years mirror the correlations that we previously reported between structural connectivity at 4–6 years of age and language scores for EPT children with a history of language difficulty, delay or disorder.^74^ The cerebellum is increasingly being recognized as being important for language development in preterm children, term children and preterm adults.^27,85-89^ The negative associations here might seem surprising in light of this literature. However, we previously suggested that EPT children at 4–6 years of age rely on this pathway to compensate for brain dysmaturation or injury, in accordance with reports that the cerebellum is important for positive adaptability, adjusting brain pathways supporting language and motor performance after perturbation.^25,74,86,90^ The negative associations with cerebellar connectivity here might reflect a heterogeneity in language ‘phenotypes’, as it is not yet known which of the 2-year-olds in the current study will go on to develop language difficulty, delay or disorder at school age and which will not. In fact, assessment of language at 2 years of age has been shown to poorly predict later language functioning in prematurity.^39,41^ Later assessments of language development are more valid.

Additionally, one could conclude that posterior and cerebellar connectivity we are seeing at 4–6 years in well-performing EPT children are related to post-discharge factors, which stabilize the developing—but atypical—language network. In this context, the contrast in our results from infancy and from early childhood could reflect a critical period of development in cerebellar networks. Cerebellar volume increases 5-fold from 24 weeks of gestation to TEA in humans, experiencing significant growth in the third trimester that appears to be sensitive to the perinatal and neonatal environment in human and animal models.^27,91^ The earlier one relies on a cerebellar ‘booster’ for language skills, the more likely one might be to need this cerebellar booster or stabilizer due to dysmaturation elsewhere. One could speculate that the less EPT infants at TEA have to rely on this alternate pathway, the better performance at 2 years, but—of those babies who have to rely on this alternate pathway through development—those who have the most robust tracts within this pathway at 4–6 years perform best. Future studies of language development in EPT should focus on brain connectivity from infancy through preschool, a dynamic period of not only language development but also white matter development.

Taken collectively, the structural connectometry results suggest a reprioritization of white matter during development. At 4–6 years, stabilization of the atypical, extracallosal pathway and prioritization of posterior and cerebellar pathways appear to be advantageous, but reliance on these pathways at TEA appears to be disadvantageous in the results we report here. These findings are congruent with a body of scientific evidence from humans and animal models that preterm birth results in neural dysmaturation.^92^ There is evidence for this dysmaturation of multiple organ systems in the context of prematurity, including human and animal models.^92,93^ In the brain, this is likely due not only to the stress inherent in completing the third trimester of neural development outside of the womb but also to interventions that confer an increased chance of survival, such as antenatal corticosteroids (with the trade-off being developmental hypermaturation, dysmaturation or arrest).^94-96^

In our investigation of white matter properties using CSD with CBFE, we were unable to generate any findings that were predictive of later language performance in infants born EPT. This is in contrast to previously published results relating CSD with CBFE to later outcomes in infants born VPT (<32 weeks completed gestation) from the same CINEPS cohort.^81,97^ Those results focused on motor^81^ and vision outcomes^97^ and/or include more infants; thus, we suspect this is due to lack of statistical power after controlling for multiple comparisons. However, discordant results using connectometry and fixel-based analyses could be due to several methodological differences in both the reconstruction of the diffusion signal, tractography method and statistical approach. While both are based on higher-order diffusion methods meant to resolve accurate fibre architecture, including crossing fibres, and quantifying white matter health in high *b*-value, high directional dMRI data, each uses a different technique to estimate the diffusion signal with respective pros and cons.

Connectometry utilizes GQI, a model-free method similar to DTI, which attempts to directly estimate the SDF of the signal and requires only one parameter to be set (i.e. the diffusion sampling length).^63^ This method is not as sensitive to small fibre crossings or white matter microstructure properties but is robust to noise and generalizable across a wide range of diffusion acquisition schemes.^98^ CSD is a model-based approach, like NODDI, that aims to quantify white matter microstructure properties (e.g. FD) by modelling the diffusion signal in a voxel in multiple compartments. While this method is more sensitive to underlying fibre orientation, it is also more computationally intensive and requires high directional data (at least 45 directions) with high *b*-values (*b* = 3000) and high signal-to-noise ratio to calculate optimal results.^65,99^ Multicompartment models in neonates can only be fit optimally with a *b*-value of 2600 m/s^2^ because the compartments cannot be reliably distinguished at lower *b*-values, which may slightly skew metrics for lower *b*-values.^99,100^ However, reasonable results have still been obtained at lower *b*-values (*b* = 1000).^101^ Overall, which approach is selected for a given application depends on two key points: (i) GQI better quantifies white matter macrostructure (i.e. QA/connectivity) while CSD is aimed more towards quantifying white matter microstructure, and (ii) GQI is more likely to produce false negatives (i.e. specificity > sensitivity) while CSD is more likely to produce false positives (sensitivity > specificity) at the individual level.^98^ Additionally, our investigation of preferred preprocessing pipelines for each method (TORTOISE and MRtrix-specific) reveals that the choice of preprocessing affects this trade-off. Because the results were more widely distributed in the white matter but had a higher *P*-value for the MRtrix preprocessing steps compared to the TORTOISE pipeline, this suggests that preprocessing through TORTOISE has better specificity than sensitivity while the MRtrix-specific pipeline has better sensitivity than specificity (Supplementary Figs 1 and 2).

After the diffusion signal is reconstructed, both methods use diffeomorphic nonlinear warping to align a participant’s data to a common template and apply the diffeomorphic mapping to the SDFs/FODs.^62,102^ The metrics used for further analyses differs slightly for each method and is worth noting. Both use the amplitude of each SDF/FOD ‘peak’ to quantify the signal for a given fibre direction, but due to the difference in reconstruction methods, the interpretation differs. In connectometry, QA is used and is a measure of the diffusivity and density of the underlying water in a voxel.^103^ In fixel-based analyses, FD is used and is approximately proportional to the intracellular volume in the underlying axons.^104^ Importantly, in prior studies limited to adult participants, FD is only specific to intracellular white matter volume at *b* ≥ 3000 mm/s^2^.^104^ At lower *b*-values, extracellular space outside of axons contribute. QA, on the other hand, does not give information of the fibre or axonal density but is reliable and consistent across *b*-values. Of course, increasing *b*-value and/or number of directions will improve the estimate, but it is not necessarily able to correctly interpret the metric. Our dMRI data are relatively high directional but acquired at a moderate *b*-value (2000 mm/s^2^) and prone to noise, a commonly encountered scenario in infant scanning.^105-107^ It is reasonable to assume that QA is a more consistent metric across participants, which may provide more statistical power.

Our study has several limitations with the biggest being the modest sample size. This is due to the restrictive nature of our research question (limiting to children born EPT without clinical diagnosis of significant brain injury). For this analysis, single shell data (b2000) was used to increase the number of participants included in both analyses. We did not include the b800 data, because shells below b1000 may make the recovered FODs less accurate. This is a limitation of our study. However, our group previously reported significant associations with vision and cerebral palsy outcomes using CSD applied to only b2000 data, as we have here.^81,97^ Additionally, language scores assessed with the BSID at 2 years corrected age are not always predictive of language scores assessed in later childhood or in adulthood. This highlights the sensitivity of testing at different ages with different batteries and the need for longitudinal studies such as ours to follow EPT children with serial neuroimaging and standardized assessments to describe the trajectory of language development and associated brain networks and identify critical periods in neurodevelopment. We only have neuroimaging at a single time point. Future studies should try to analyse changes over time at the individual and group levels.

Despite these potential limitations, our study has several strengths, including the prospective longitudinal nature of the study. Additionally, the diffusion data we have obtained are high-angular-resolution data especially amenable to tensor-free analyses and tractography analyses. We use two sophisticated higher-order analytical techniques that are more sensitive to crossing fibres to interrogate the white matter structure and connectivity of the brain in a data-driven way. This represents the first implementation of structural connectometry in DSI Studio for preterm infants, to our knowledge, and we set out to compare these findings to CBFE to provide a complementary, more comprehensive view into the developing preterm brain.

Our findings provide new information about the ontogeny of structural brain networks supporting language in extreme prematurity. We used two higher-order approaches to characterize white matter in extreme prematurity and correlate it with later language outcome. Structural connectometry analysis of dMRI obtained at TEA significantly related to language performance at 2 years corrected age in this cohort of children born EPT. However, unlike our previously published findings in older EPT children, cerebellar connectivity at TEA is negatively correlated with later language scores.^25,74^ This could point to the importance of post-discharge factors in promoting connectivity in the cerebellar white matter to stabilize or ‘boost’ the developing language networks in prematurity.

## Supplementary Material

fcae126_Supplementary_Data

## Data Availability

The data that support the findings of this study are available from the corresponding author upon reasonable request.
