# Supplementary material for: Structural connectivity at term equivalent age and language in preterm children at 2 years corrected
Source: Brain Commun. 2024 Apr 10;6(2):fcae126. doi: 10.1093/braincomms/fcae126 (PMC11043656; doi:10.1093/braincomms/fcae126)

Supplementary Material

Supplementary Table 1: Demographic, Clinical, and Neurodevelopmental Scores for EPT Infants with Complete versus Incomplete Imaging. There were no significant differences between those groups in our outcomes (BSID scores). However, there were differences between children who had complete neuroimaging versus those who did not have complete neuroimaging in sex, race, and global brain abnormality score. Infants who had complete neuroimaging that passed quality control were more likely to be female (p=0.02) and of White/Caucasian race (p=0.001) with slightly lower global brain abnormality scores versus the group who did not have imaging that passed quality control (6.2 vs. 8.5, p=0.02).

Supplemental Table: Demographic, Clinical, and Neurodevelopmental Data (n=95)

		Imaging (n=54)	No Imaging (n=41)	p value
**Gestational Age (Weeks)**		26.36	26.03	0.24
**Birth Weight (Grams)**		871.59	851.78	0.66
**BSID Language Composite**		91	90	0.9
**BSID Cognitive Composite**		90	86	0.08
**BSID Motor Composite**		91	89	0.4
**Global Brain Abnormality Score**		6.24	8.51	0.02*
**Postmenstrual Age at MRI (Weeks)**		43.07	42.76	0.23
**Sex**	Female	31	13	0.02*
	Male	23	28	
**Race**	American Indian/Alaskan Native	1	0	0.001*
	Black/African American	4	15	
	White/Caucasian	39	18	
	Multiple/Other	5	7	
	Decline to Respond	5	1	
**Mode of Delivery**	Breech	1	0	0.32
	C-Section	33	30	
	Vertex	20	11	
**Small for Gestation**	SGA	4	2	0.7
**Antenatal Corticosteroids**	ANCS	51	40	0.63
**Antenatal Magnesium**	Magnesium	48	40	0.23
**High Risk Social Index**	High Risk Social	7	9	0.28
**Intraventricular Hemorrhage**	Mild IVH	9	8	0.79
**Maternal Hypertension**	HDP	19	14	1
**Necrotizing Enterocolitis**	NEC	3	4	0.46
**Neonatal Sepsis**	Sepsis	12	12	0.48
**Patent Ductus Arteriosus**	PDA	27	20	1
**Postnatal Caffeine**	Caffeine	54	41	1
**Severe Bronchopulmonary Dysplasia**	Severe BPD	18	22	0.06
**Severe Retinopathy of Prematurity**	Severe ROP	9	8	0.79

Note: Variables with p<0.05 denoted by *. Continuous data presented as Mean. Categorical data presented as Count. Continuous data between groups compared using independent samples t test. Categorical data compared using Fisher's exact test. ANCS = Antenatal Corticosteroids. BPD = Bronchopulmonary Dysplasia. BSID = Bayley Scales of Infant Development. C-Section = Cesarean Section. HDP = Hypertensive Disorders of Pregnancy. IVH = Intraventricular Hemorrhage. MRI = Magnetic Resonance Imaging. PDA = Patent Ductus Arteriosus. ROP = Retinopathy of Prematurity. SGA = Small for Gestational Age.

Supplementary Figure 1: Structural Connectometry in Extremely Preterm Infants at Term-Equivalent Age (Preprocessed using MRtrix/FSL-EDDY). Results of connectometry analysis relating white matter connectivity at term-equivalent age (TEA) to language composite scores from the Bayley Scales of Infant and Toddler Development, 3rd Edition (BSID) assessed at 2 years corrected age. Analyses preprocessed using the MRtrix/FSL pipeline revealed tracks negatively (middle row, FDR-p = 0.008) associated with BSID language scores while controlling for multiple comparisons and for the following confounding variables: PMA (post-menstrual age at time of MRI); sex; social risk score; and global brain abnormality (GBA) score (T threshold = 2.5, Length threshold = 20 voxels, 2 pruning iterations, 4000 permutations). The top row is a figure color-coded by fiber orientation/direction, and the bottom row is color-coded by local t-statistic value of the underlying SDFs. Tracks negatively associated with language performance include bilateral corticospinal tracts (CST), middle cerebellar peduncle, bilateral cerebellar tracts, posterior IFOF, and bilateral uncinate fasciculi. Notably, all tracks shown must have first passed the t=2.5 threshold for the connectometry analysis. T-score here is for the subsequent analysis after these tracks were selected. Strongest effects for tracks negatively associated with language are in the middle cerebellar peduncle, and left CST and corticostriatal tracts.

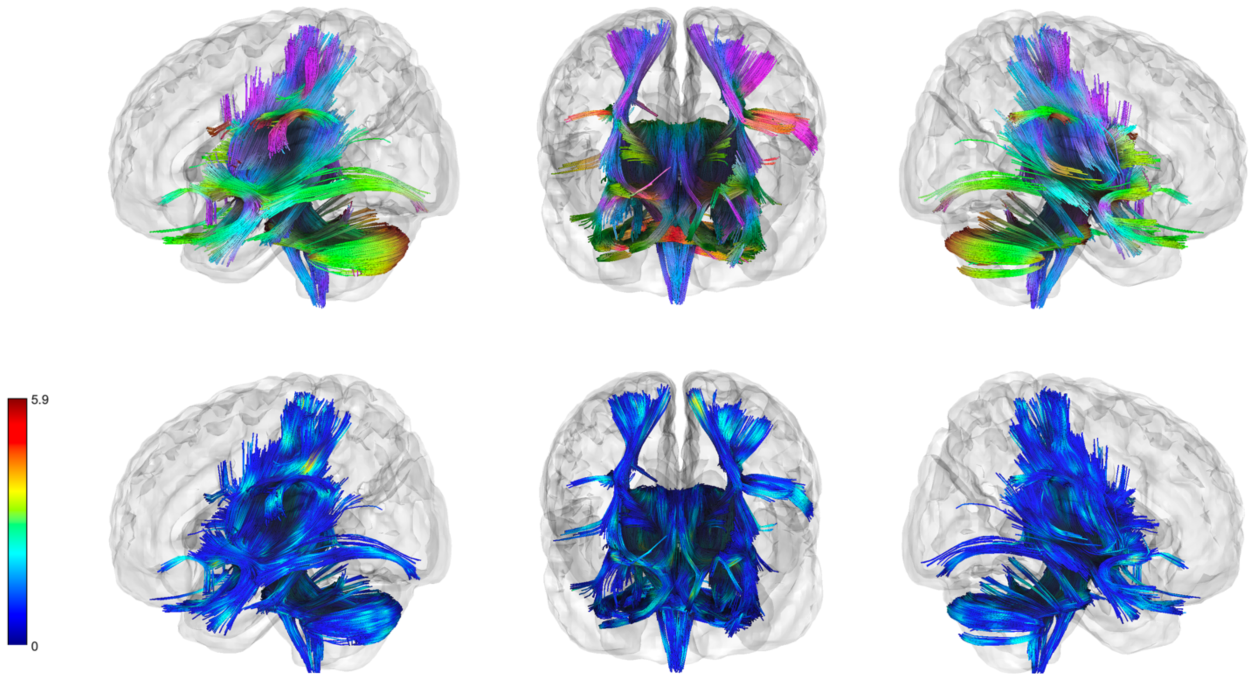

Supplementary Figure 2: Language Scores Plotted by Mean Normalized Quantitative Anisotropy (NQA) Residuals (Preprocessed using MRtrix/FSL-EDDY). Plots of BSID Language Scores by Mean NQA values, adjusted for the partial effects of sex, global brain abnormality score, social risk score, and PMA, for tracks negatively associated with BSID Language in the connectometry analysis with preprocessing performed using MRtrix. Correlation coefficient (Spearman Rho) between adjusted mean NQA and BSID Language is also shown. Tracks negatively associated with language performance show the most robust effect in the bottom half of NQA values.

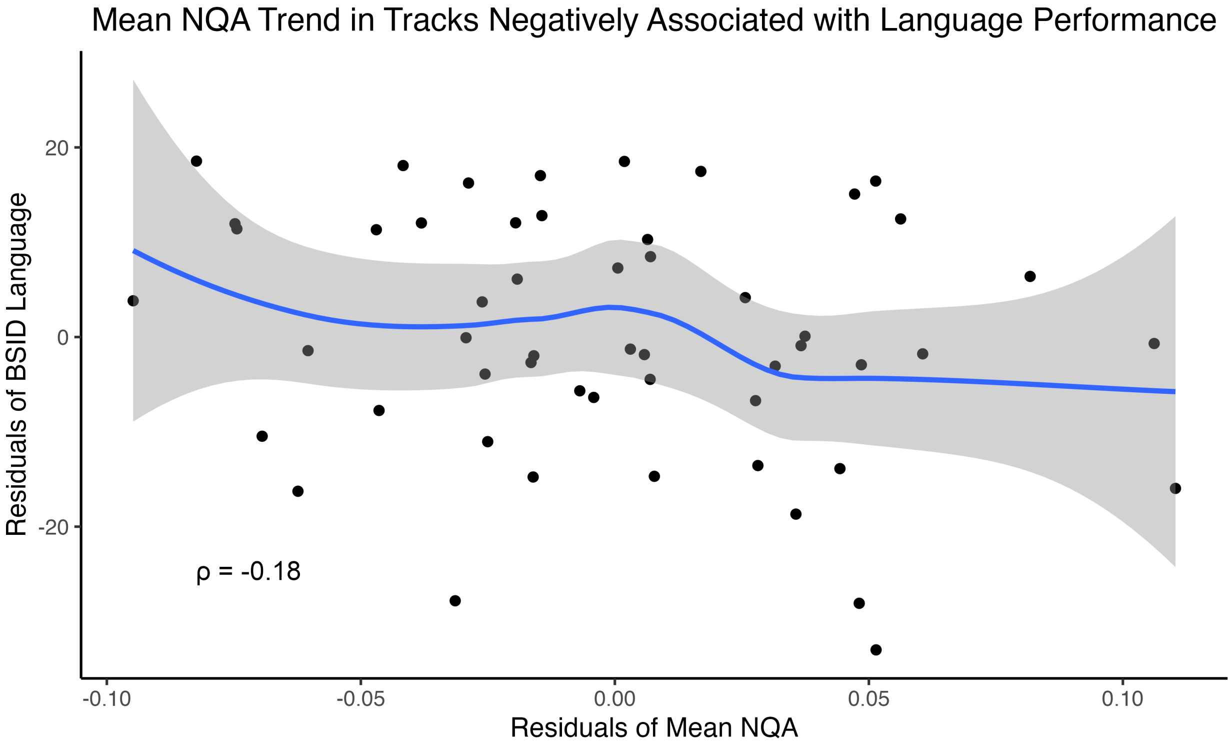

Supplement: fcae126_Supplementary_Data [file fcae126_supplementary_data.pdf]
